# Concepts and Terms in Genetic Research—A Primer

**Published:** 2002

*It is further remarkable that drunkenness resembles certain hereditary . . . diseases*.

—Benjamin Rush, *Inquiry into the Effects of Ardent Spirits upon the Human Body and Mind*, 1785

People have long suspected that alcoholism has a genetic component. Research over the last few decades has indicated that 40 to 60 percent of the susceptibility to alcoholism is inherited. Understanding the genetic mechanisms of addiction vulnerability is therefore one of the highest priorities of today’s alcohol research. The knowledge base in this area is increasing exponentially, fueled by advances in genetic technology and data analysis. This sidebar reviews some basic genetic concepts mentioned in this issue of *Alcohol Research & Health* and tries to place them in a scientific and historical context. This discussion is offered with the caveats that traditional genetic concepts are changing so rapidly that most print and many online data sources rapidly are becoming obsolete, and this review—as well as many of the definitions in the accompanying glossary—is greatly simplified and subject to numerous exceptions.

## DNA: The Molecule of Life

All the genetic information needed to sustain life is encoded in a long, threadlike molecule called deoxyribonucleic acid (DNA), which makes it possible to transmit this information from one generation to the next. A single strand of DNA is composed of a chain of building blocks called nucleotides. Each nucleotide consists of two subunits: (1) a modified sugar molecule (i.e., deoxyribose phosphate) and (2) one of four molecules known as organic bases called adenine (A), thymine (T), guanine (G), and cytosine (C). Sequential nucleotides are held together by strong chemical bonds between adjacent sugar phosphate subunits, leaving the bases exposed.

DNA typically occurs as a pair of nucleotide strands that are intertwined to form a double helix, a three-dimensional configuration resembling a spiral staircase. The sugar phosphate backbones form the outside of the helix. The bases within the helix are joined together by relatively weak chemical bonds, forming the steps of the “staircase.” Strict rules govern the formation of base pairs between two DNA strands. Because of their chemical structure, adenine always binds to thymine, and guanine always binds to cytosine. Bases that can bind to each other are called *complementary;* similarly, the two strands of a DNA double helix also are complementary (see figure). The principle of complementarity is the basis of DNA’s ability to store biological information and pass it on from generation to generation.

**Figure:**
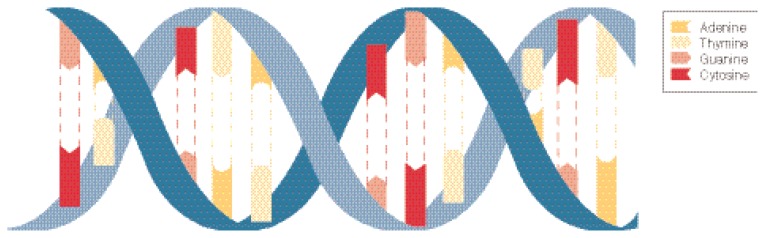
DNA includes four types of nucleotides: adenine, thymine, guanine, and cystosine. Adenine always bonds with thymine, and guanine always bonds with cytosine. These two combinations form the double-stranded DNA molecule.

The DNA of most organisms is swaddled by protein molecules and tightly packaged into larger structures that during a certain phase of the cell cycle can be visualized as rod-shaped chromosomes. Chromosomes vary in size and shape and occur in matched pairs inside the nucleus of almost every cell of the body. The number of chromosomes per cell depends on the organism; humans, for example, have 23 pairs. When body cells proliferate, their chromosomes duplicate before the cell divides, ensuring that each daughter cell will receive a complete set of paired chromosomes. Reproductive cells, by contrast, are produced by a specialized type of cell division that distributes only one member of each chromosome pair to each egg or sperm cell. When an egg and a sperm cell fuse during fertilization, their chromosomes combine so that the developing offspring contains a full set of chromosomes with an equal share of genetic material inherited from each parent.

## Proteins and the Genetic Code

Proteins are the basic structural and functional molecules of living things. As physical components of the cell’s architecture, they not only contribute to an organism’s basic form (i.e., are structural proteins), but simultaneously fulfill a myriad of functional roles. For example, nonstructural proteins play major roles in biochemical and metabolic events within cells; carry messages between cells; or circulate through the entire body via the bloodstream, functioning as hormones, immune system components, and transporters of oxygen and other vital substances. The most important proteins (at least within the context of this sidebar) are those that serve as enzymes. Whether they move freely in the cell or are attached to a cell structure, enzymes initiate and facilitate almost all of the countless chemical reactions that sustain life.

Proteins are composed of chains of up to several thousand subunits called amino acids. Twenty different amino acids participate in protein formation. The sequence of the amino acids in a protein determines its function. Some researchers have speculated that the human body can produce up to 1 million different proteins on an as-needed basis, although fewer than 100,000 are likely to be present in a cell at any given time.

The sequence of amino acids in a protein is determined by the sequence of nucleotide bases in the stretch of DNA that encodes the protein. Each amino acid is represented by a sequence of three DNA bases, called a triplet. Because 4 different bases can be combined into 64 different triplets, most of the 20 amino acids are represented by more than 1 triplet. (In fact, only 61 triplets code for amino acids; the remaining 3 serve as “stop signs” indicating the end of a protein’s amino acid sequence.) It took more than a decade of research before scientists finished breaking the genetic code—that is, matching every triplet to its corresponding amino acid. The same genetic code is shared by every organism on the planet. For example, the DNA nucleotide combination A-T-G (adenine-thymine-guanine) always codes for the amino acid methionine, regardless of where it may appear within the overall DNA coding sequences of different proteins.

## DNA at Work: Gene Expression and Protein Synthesis

The term “gene expression” is variously defined; in this section it is used to refer to all processes involved in DNA-directed protein synthesis. Because the chromosomes containing the DNA are sequestered in the nucleus, whereas protein synthesis occurs at cytoplasmic structures called ribosomes, gene expression involves the use of small helper molecules that relay the genetic information from one cell structure to the other. These molecules are composed of ribonucleic acid (RNA). RNA is identical to DNA in all but two respects: (1) ribose replaces deoxyribose in the sugar phosphate subunit of each RNA nucleotide; and (2) thymine is replaced by uracil (U), which, however, also binds (i.e., is complementary) to adenine. Three families of RNA molecules play prominent roles in gene expression (as described below)—messenger RNA (mRNA), transfer RNA (tRNA), and ribosomal RNA (rRNA).

During the first stage of gene expression, which is called transcription, an mRNA molecule is produced that is an exact copy of the relevant DNA region. To produce this molecule, the DNA double helix untwists temporarily, exposing the base sequence that encodes the protein to be synthesized. One of the two DNA strands serves as a template for the formation of the mRNA, which is generated with the help of a host of enzymes and regulatory molecules. As in the DNA, three-base sequences specify which amino acids will later be used when the protein is assembled; these RNA triplets are called codons. (It is important to note that, by convention, it is the mRNA codons rather than their complementary DNA triplets that are considered the genetic code. For example, the genetic code for methionine is T-A-C, corresponding to the DNA triplet A-T-G as described in the previous section.) The initial mRNA molecules undergo further processing in the nucleus to eliminate extraneous sequences—a process called splicing—before the finished molecules migrate from the nucleus to the ribosomes.

The second stage of protein synthesis is called translation; it involves all three major forms of RNA. The mRNA molecules attach to ribosomes, which are composed of rRNA and protein. Meanwhile, amino acids attached to tRNA aggregate near the chromosomes. The three-dimensional structure of each tRNA molecule includes a site at one end that binds to a particular amino acid, and a base sequence at the other end that recognizes the mRNA codon specific for that same amino acid. During translation, tRNA molecules with their attached amino acids are recruited to the ribosomes in the order specified by the sequence of mRNA codons. With the help of multiple enzymes and regulatory molecules, the amino acids are then detached from their tRNA carriers and linked to each other to form the desired protein.

## Genes

The reader has undoubtedly noted the conspicuous absence of the word “gene” from the preceding text. The decision to postpone discussion of the term was motivated by the fact that there is no single definition of the term “gene.” For example, the Cambridge Health Institute’s online database entitled “Gene Definitions” (http://www.genomicglossaries.com/) contains several pages of unembellished alternative definitions for “gene.” The complexity results from the ever-increasing, and changing, understanding of how the genetic information encoded in the DNA is converted into the myriad of proteins found in each cell of the organism. The following discussion is further evidence of the complexity of this topic.

In the early days of genetic research, starting around the beginning of the 20th century, genes were presumed to be some form of “units” located linearly along the chromosome like beads on a string. Each individual gene was presumed to be responsible for the synthesis of one particular protein. Extensive research from approximately 1950 to the early 1980s filled in the details of DNA structure and function described earlier in this review. Briefly stated, a gene was defined as a linear sequence of DNA nucleotide bases that specify the instructions for making a particular protein.

New discoveries based on the explosive proliferation of functional genomic technology (see the discussion of genomics below) in the 1970s resulted in extensive reassessment of these classical genetic concepts. More detailed analyses of DNA found that, for example, genes can reside within one another, share some of their DNA sequences, contain sequences that are not represented in the final sequences (i.e., noncoding sequences), and are transcribed and spliced in complex patterns. Furthermore, genes that are similar in sequences (i.e., belong to the same sequence family) can overlap in function. In addition, mRNA molecules are not just simple copies of the DNA that are faithfully translated into proteins. Instead, newly transcribed mRNA molecules can be cut, pasted, rearranged, inactivated, or simply degraded. Finally, the one gene–one protein hypothesis also no longer holds true. Thus, a gene may be spliced in different ways to produce several distinct proteins; alternatively, a protein may be a product of several adjacent genes.

As a result of all of these discoveries, multiple definitions for “gene” now exist, each of which has validity in a different context. This situation tends to impede communication between researchers across subdisciplines, and some scientists have even proposed abandoning the term altogether. Because the term “gene” is being used so much and has been associated so tightly with the genetic knowledge base to date, however, a more appropriate goal might be to keep refining its definition and promote its accurate use (Judson 2001). For the purpose of this journal issue, a gene can most easily be defined as a combination of DNA segments that together are capable of generating one or more functional proteins. These segments include coding sequences (i.e., exons), noncoding sequences within the gene (i.e., introns), regulatory sequences outside or within the gene, and nucleotide sequences that indicate where transcription should begin and end.

## Genetic Variability and Why There Is No Gene for Alcoholism

According to geneticist Horace Freeland Judson (2001), “The phrases current in genetics that plainly do most violence to understanding begin ‘*the gene for’”* (p. 769). This statement reflects the fact that it is incorrect to say that a person “inherits the gene” for a disease, as all humans generally carry the same number and types of genes. However, many genes exist in different variants, or alleles, and individuals can inherit different alleles, making each person genetically unique. The mixture of specific alleles that contribute to a particular person’s size, shape, personality, and so on, is called the genotype. (In contrast, the term “genome” refers to the total genetic material of an organism.) The observable physical or behavioral characteristics (and internal physiological differences) that result from a specific genotype constitute an individual’s phenotype.

A DNA region for which several alleles exist is said to be polymorphic. Most polymorphisms represent normal variation. For example, various alleles of a gene that affects eye color result in a person having brown or blue eyes. Sometimes, however, abnormal alleles can give rise to proteins that are inactive or function abnormally and which can contribute to disease. For example, cystic fibrosis is caused by a defect in a single gene. Genetic defects often arise from mutations, changes in nucleotide sequence resulting from exposure to x-rays or toxic substances, or from unknown causes. Polymorphisms that occur in less than 1 percent of the population are considered mutations.

Most human traits are too complex to be determined by a single gene with two or more alleles. Many traits, such as height or intelligence, vary continuously across a population and interact with environmental influences. These traits, which are called quantitative traits, are influenced by the cumulative effects of multiple genes, each of which may contribute a relatively small effect. Genes that influence a quantitative trait are called quantitative trait loci (QTLs). Virtually all behavioral characteristics are quantitative traits, including those that influence alcohol consumption and the effects of alcohol on the person. Consequently, there is no single “gene for alcoholism,” or even “allele for alcoholism.” Instead, a combination of multiple polymorphic genes, interacting with the environment, determines a person’s risk for the disease.

## Genomics and Proteomics

Each cell must produce a specific set of proteins to fulfill its functions, and protein synthesis often must be adjusted in response to changing internal and external events. Accordingly, gene expression is regulated by an assortment of proteins and other substances that tell genes in different cells when to turn on and off. As a result, only a fraction of the estimated 35,000–40,000 genes present in each cell is switched on at any given moment, producing no more than 6,000 primary translation products. Nevertheless, the complete set of proteins that can be produced in an organism (i.e., the proteome) is considerably larger and more complex than the organism’s genome. In fact, whereas genomes are relatively static, proteomes are constantly changing. Some researchers believe that the number of different protein molecules expressed by the human genome may be closer to 1 million than the standard estimate of 100,000.

Genomics and proteomics—the comprehensive study of the structures, functions, and interactions of whole sets of genes and proteins, respectively—represent attempts to look at gene function and protein interactions globally and dynamically. Researchers have discovered literally hundreds of mechanisms to explain the discrepancy between the size of the genome and that of the proteome. For example, as mentioned earlier, the protein-coding segments of a gene—the exons—are separated by noncoding sequences—the introns. Some introns help regulate gene expression; the function of others still is unknown. During gene expression, the developing mRNA strand first contains both introns and exons. The introns are then removed from the mRNA and the exons are spliced together, leaving an uninterrupted series of coding sequences for translation. However, the initial transcript sometimes can be spliced in different ways, thereby increasing the number of different proteins that can be produced by a single gene. In addition, gene expression can be modified after translation. Such post-translational modifications (PTMs) include (among others) alternative protein splicing; deletion of amino acid sequences; chemical modification of specific amino acids; and the attachment to the protein of small chemical and other biological molecules, ranging in size from small chemical fragments (e.g., phosphate groups as in DNA and RNA) to entire molecules (e.g., sugar molecules or other proteins). Again, these modifications can lead to the generation of more than one protein from one gene.

## Conclusions

The preceding discussion shows the complexity of the genetic basis of alcoholism, which probably involves numerous genes, each of which has only a relatively small influence. To identify those genes and their contributions, it is crucial that researchers use both “traditional” genetic approaches that study individual genes and their functions and more recent genomic and proteomic approaches which allow for the examination of the structures and functions of whole gene or protein systems. Combining these approaches should help researchers gain a better understanding of the genetic underpinnings of alcoholism and other complex disorders.

The articles in this issue of *Alcohol Research & Health* show how researchers are expanding the range of genetic tools at their disposal and, using these tools, are making great strides in elucidating the roles of various genes in shaping a person’s risk for alcoholism. More extensive use of these cutting-edge tools is likely to lead to additional progress in the diagnosis, prevention, and treatment of alcoholism.

The following glossary of common genetic terms, although far from being comprehensive, is intended to give readers a better understanding of the terminology used throughout this issue of the journal. For more information on these terms and the concepts discussed in this article, readers also are referred to the suggested reading list that follows.

## GLOSSARY

Allele: One of two or more variants of a *gene* or other *DNA* sequences. Different alleles of a *gene* generally serve the same function (e.g., code for a *protein* that affects eye color) but may produce different *phenotypes* (e.g., blue eyes or brown eyes). Some alleles may be defective and produce a *protein* that has no function or an abnormal function.
Amino acids: A class of biological molecules, 20 of which serve as building blocks of *proteins*.
Association studies: Population-based genetic studies that examine whether an *allele* of a certain *gene* or *marker* co-occurs with a *phenotype* (e.g., a disease) at a significantly higher rate than predicted by chance alone.
Base: In genetics, that portion of a *nucleotide* molecule that contributes to the *genetic code. DNA* bases include adenine, thymine, guanine, and cytosine; in *RNA*, uracil replaces thymine.
Bioinformatics: The collection, organization, storage, analysis, and integration of large amounts of biological data using networks of computers and databases.
Candidate gene: A *gene* that has been implicated in causing or contributing to a particular *phenotype* (e.g., a disease).
cDNA (complementary DNA): A segment of *DNA* identical in base sequence to at least part of the coding sequence of a *gene*, generated in the laboratory from a natural *mRNA* molecule. cDNA can be produced in large quantities for sequencing and other genetic studies.
Chromosomes: Microscopic rod-shaped structures composed of double-stranded *DNA* and *proteins;* can be visualized during a certain phase of the cell cycle; generally found within the cell nucleus. Chromosomes are often regarded as representing the entire *genome* of an organism.
Cloning: The production of multiple exact copies of a single *gene* or other segment of *DNA* to obtain enough material for further study. Also applied to the production of complete, genetically identical animals.
Codon: A sequence of three consecutive *nucleotide base* subunits in an *mRNA* molecule that together represent the *genetic code* for a particular *amino acid*.
Cytoplasm: The entire contents of a cell apart from the nucleus.
DNA (deoxyribonucleic acid): The molecule that carries the *genetic code* in all organisms except some viruses. DNA is composed of a linear sequence of *nucleotides*.
Endophenotype: A heritable trait or characteristic that is not a direct symptom of the condition under investigation but has been shown to be associated with the condition; for example, certain neurobiological characteristics have been noted in people with alcoholism and may be used as endophenotypes to identify people at risk for alcoholism.
Enzyme: A substance (usually a *protein*) that speeds up, or catalyzes, a specific biochemical reaction without being itself permanently altered or consumed.
Exon: A sequence of *nucleotides* within a *gene* that contains the information for part or all of the *gene* product (also see *intron*).
Expressed sequence tag (EST): A unique segment of *cDNA* with a *base* sequence identical to at least part of the coding region of a *gene*, generally used as landmark for mapping.
Fraternal twins: Twins that have been produced by the simultaneous fertilization of two egg cells and who therefore share only on average 50 percent of their *genes*, just like other siblings; also called dizygotic twins.
Gene: A combination of *DNA* segments that together constitute a unit capable of expressing one or more functional gene products. The segments of a gene can include *exons, introns*, regulatory regions, and *nucleotide* sequences that indicate where *transcription* should begin and end.
Gene-based therapy: Any treatment regimen that uses or targets genetic material. For example, one can attempt to insert a healthy *gene* to correct a defect resulting from a disease-associated *allele* or to implant cells whose genetic makeup is modified so they may produce products of therapeutic benefit.
Gene expression: The process of converting the genetic information encoded in *DNA* into a final gene product (i.e., a *protein* or any of several types of *RNA*). Because changes in cellular protein synthesis are often estimated by measuring *mRNA* levels, the term “gene expression” is often misleadingly used as synonymous with *transcription*. However, gene expression encompasses *transcription*, processing and *splicing* of the *mRNA*, *translation*, and *post-translational modification* of the *protein* product.
Gene mapping: Determination of the positions of *genes* on a *chromosome* relative to one another.
Gene regulation: The process through which the cell determines—through interactions among *DNA, RNA, proteins*, and other substances—when and where *genes* will be activated and how much gene product will be produced.
Genetic code: The way in which the information carried by the *DNA* molecules determines the arrangement of *amino acids* in the *proteins* synthesized by the cells. Each of the 20 *amino acids* found in proteins is represented by 1 or more units of 3 consecutive *nucleotide bases* (i.e., *codons*) in the *mRNA* and in the *DNA* from which the *mRNA* is derived. All living organisms and viruses use the same genetic code.
Genetic recombination: The exchange of corresponding *DNA* segments between adjacent *chromosomes* during the special type of cell division that results in the production of egg and sperm cells. Genetic recombination can result in new combinations or arrangements of *alleles*.
Genome: The total genetic material of an organism or species.
Genomics: The comprehensive study of the interactions and functional dynamics of whole sets of *genes* and their products.
Genotype: The genetic makeup of an individual organism that is determined by the specific *alleles* of each *gene* carried by the individual. Differences in *alleles* among individuals interact with environmental influences to account for the differences in *phenotype* observed among those individuals.
Identical twins: Twins that have been produced by the division of a single fertilized egg cell and who therefore have an identical *genotype;* also called monozygotic twins.
Inbreeding: The process of repeatedly mating sequential generations of sibling animals, usually mice. Within an inbred strain, same-sex animals are genetically identical and have two identical copies of each *allele* at corresponding *loci* of the paired *chromosomes*.
Intron: Noncoding *DNA* segments within a *gene*, some of which may help regulate the timing and extent of the *gene’s expression*.
Knockout: The deletion or deactivation of a *gene* in a mouse or other laboratory animal in all cells, including the germ cells, to create a line of animals that are incapable of producing the *gene* product.
Linkage analysis: The comparison of two groups of subjects (e.g., people with and without a given disease) to evaluate association between an *allele* and a *phenotype* (e.g., a disease).
Linkage disequilibrium: The tendency of *alleles* located close to each other on the same *chromosome* to be inherited together.
Locus: A specific location on a *chromosome;* also, the actual *nucleotide* sequence at that location.
Marker: A *DNA* sequence whose chromosomal location has been determined and which can be used in *linkage analyses* to track the inheritance patterns of *genes* that have not yet been identified, but whose approximate locations are known; markers typically have multiple *alleles*.
Microarray technology: An automated, high-throughput technique for simultaneously analyzing thousands of different *DNA* sequences or *proteins* affixed to a thumbnail-sized “chip” of glass or silicon. *DNA* microarrays can be used to monitor changes in the expression levels of *genes* in response to changes in environmental conditions or in healthy versus diseased cells. *Protein* arrays can be used to study *protein* expression, *protein–protein* interactions, and interactions between *proteins* and other molecules.
mRNA (messenger RNA): A type of *RNA* that relays the coding information for *proteins* from the *DNA* in the nucleus to the *ribosomes* in the *cytoplasm*, where actual *protein* synthesis occurs.
Mutation: A heritable change in the *DNA nucleotide* sequence that can potentially result in a change in the function of one or more *genes*.
Nucleotides: Biological molecules with a variety of physiologic and metabolic functions that also serve as the building blocks of *DNA* and *RNA*.
Pharmacogenomics: The study of the effect of an individual’s *genotype* on the body’s potential response to medications.
Phenotype: The observable structural or functional characteristics of an individual organism that result from the interaction of its *genotype* with environmental factors.
Physical mapping: Determining the specific physical locations of *genes* and *markers* on each *chromosome*, with the distances between them expressed in units that quantify the actual amount of *DNA* between two *loci*.
Point mutation: A change in a single *nucleotide* of the *genome*, occurring in 1 percent or less of the population. Also see *single-nucleotide polymorphism*.
Polymorphism: The presence of two or more *alleles* of a *gene* or other *DNA* sequence in a population. A variant *allele* that occurs in less than 1 percent of the population is considered a *mutation*.
Post-translational modification (PTM): The modification of a newly formed *protein;* may involve the deletion of *amino acids*, chemical modification of certain *amino acids*, or addition of small molecules (e.g., phosphate groups or sugars) to certain *amino acids*.
Promoter: A *DNA* segment located at the start of a *gene’s* coding sequence that provides a binding site for the *enzymes* that initiate *transcription*.
Protein: A large molecule composed of one or more chains of *amino acids* in a specific order.
Proteome: The totality of all the *proteins* in an organism.
Proteomics: The large-scale analysis of the structure and function of *proteins* as well as of *protein–protein* interactions.
Quantitative trait: A *phenotypic* trait that varies along a continuum within a population (e.g., height). Such traits are determined by the cumulative interaction of multiple *genes* and their *alleles* (each of which by itself has a relatively small effect on the trait) in combination with environmental factors.
Quantitative trait locus (QTL): A *polymorphic* site on a *chromosome* containing *alleles* that differentially influence the expression of a *quantitative trait*.
Recombinant inbred (RI) strain: A line of genetically identical animals produced by mating successive generations of sibling animals initially descended from the offspring of a cross between two distinct *inbred* strains.
Regulatory gene: A *gene* encoding a *protein* that plays a role in controlling the activity of other *genes*.
Ribonucleic acid (RNA): A class of molecules composed of *nucleotides*, similar to those that form *DNA*. The major types of RNA are *mRNA, tRNA*, and *rRNA*, which play important roles in *gene expression*.
Ribosomes: *Cytoplasmic* structures composed of *rRNA* and *protein*, where *proteins* are assembled from *amino acids* during *translation*.
RNA splicing: The removal of *introns* from the sequence of an *mRNA* following *transcription* to form an uninterrupted coding sequence.
rRNA: Type of *RNA* that forms structural and functional components of *ribosomes;* binds to both *mRNA* and *tRNA* to ensure the correct order of *amino acids* in a *protein* during *translation*.
Sequencing: Determining the order of *bases* in a *DNA* or *RNA* segment or of *amino acids* in a *protein*.
Single-nucleotide polymorphism (SNP): A *point mutation* that occurs in more than 1 percent of the population.
Transcription: The process by which the genetic information contained in a linear sequence of *DNA nucleotides* is converted into an exactly complementary sequence of *mRNA nucleotides;* the first stage of *gene expression*.
Transgenic animal: An animal into whose *genome* foreign *DNA* (e.g., a *candidate gene*) has been introduced to study the function of that *DNA*.
Translation: The process by which the genetic information encoded by a specific *mRNA* is converted into a corresponding sequence of *amino acids*.
tRNA (transfer RNA): A type of *RNA* molecule that carries a specific *amino acid* and matches it to its corresponding *codon* on an *mRNA* during *translation*.
